# Pacinian hyperplasia presenting with Raynaud’s phenomenon

**DOI:** 10.1080/23320885.2019.1698958

**Published:** 2019-12-13

**Authors:** Brent B. Pickrell, Simon G. Talbot, Danielle C. Costigan, Christian E. Sampson

**Affiliations:** a Division of Plastic Surgery, Harvard Medical School, Brigham and Women’s Hospital, Boston, MA, USA;; b Department of Pathology, Harvard Medical School, Brigham and Women’s Hospital, Boston, MA, USA

**Keywords:** Pacinian corpuscle, hyperplasia, CREST syndrome, Raynaud’s phenomenon, hand pain, digital ischemia

## Abstract

Pacinian corpuscle pathology is a rare clinical entity and an uncommonly reported cause of digital pain. While many prior reports implicate hand trauma, we describe a case of Pacinian hyperplasia found in a patient with Raynaud’s phenomenon and propose a potential mechanism of disease.

## Introduction

Although Pacinian corpuscles were first noted by Vater in 1741, it was Pacini who provided the first histologic description in 1835 [1]. Serving as rapidly adapting mechanoreceptors responsive to vibration and pressure, Pacinian corpuscles are pearly-gray structures that can be easily visualized without loupe magnification [2]. Macroscopically, they are ellipsoid or round with an average density of 3–5/cm^2^ in the human hand [3]. Microscopically, they consist of a single nerve fiber enclosed within a multi-laminated connective tissue capsule that gives an onion-like appearance on cross-section [3].

Pacinian corpuscle pathology constitutes a rare clinical entity and uncommonly reported cause of digital pain. Rhode and Jennings [4] were the first to classify abnormalities of the Pacinian corpuscle; this was later modified by Reznik et al. [5]. Proliferation of Pacinian corpuscles of normal or enlarged size located adjacent to the digital nerves constitutes hyperplasia [1]. While prior reports have implicated antecedent digital trauma, the mechanism behind the hyperplastic changes is yet to be fully understood.

In this report, a musician with CREST syndrome and severe Raynaud’s phenomenon was found to have Pacinian hyperplasia during digital sympathectomy.

## Case presentation

The patient is a 61-year-old right-hand dominant male pianist with CREST syndrome whose autoimmune disease course has been complicated by severe Raynaud’s phenomenon resulting in more than twenty symptomatic episodes daily. The patient underwent previous bilateral proximal palmar and wrist sympathectomies 8 years prior with some symptomatic improvement in his hand function, and this was followed by botulinum toxin injections. However, more recently, he presented to our plastic surgery hand clinic with worsening digital pain and fingertip ulcerations.

**Figure 1.**
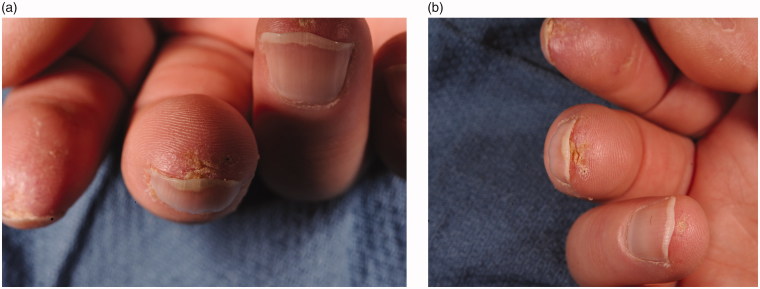
(a,b) The patient’s right index and middle fingers on preoperative evaluation showing fingertip ulceration.

On physical exam, there were exquisitely tender ulcerations at the tips of the right index and middle fingers, and left middle finger (Figure 1(a,b)). Doppler examination revealed strong triphasic signals of both radial arteries at the wrist and very weak monophasic signals of the ulnar artery at the wrist. There were strong Doppler signals at the base of each digit. Given the patient’s recurrent symptomatology and fingertip ulcerations, along with his previous therapeutic response to proximal sympathectomies, a focused distal sympathectomy at the level of the common and proper digital arteries was recommended to address his ongoing digital ischemia.

**Figure 2.**
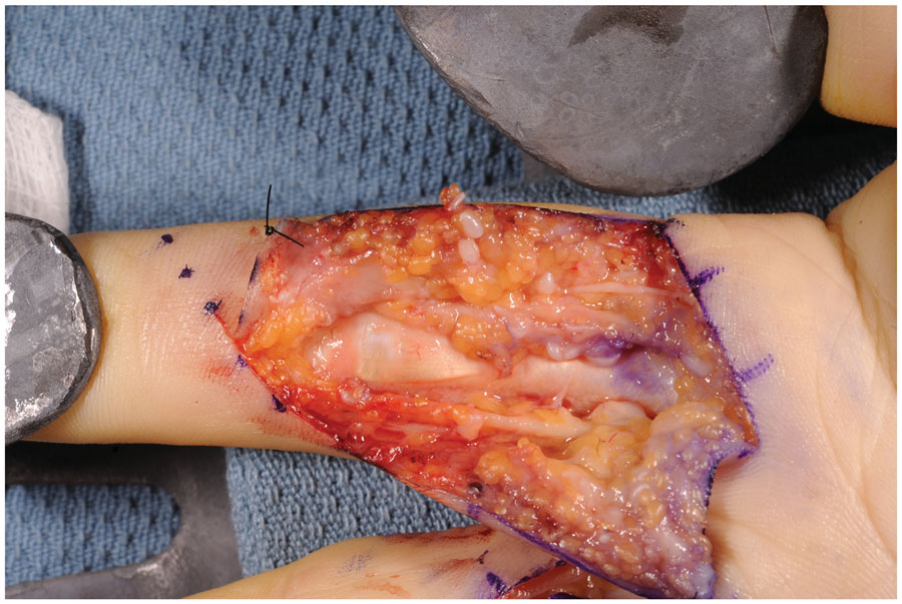
Hyperplastic Pacinian corpuscles located adjacent to the radial neurovascular bundle of the right index finger.

**Figure 3.**
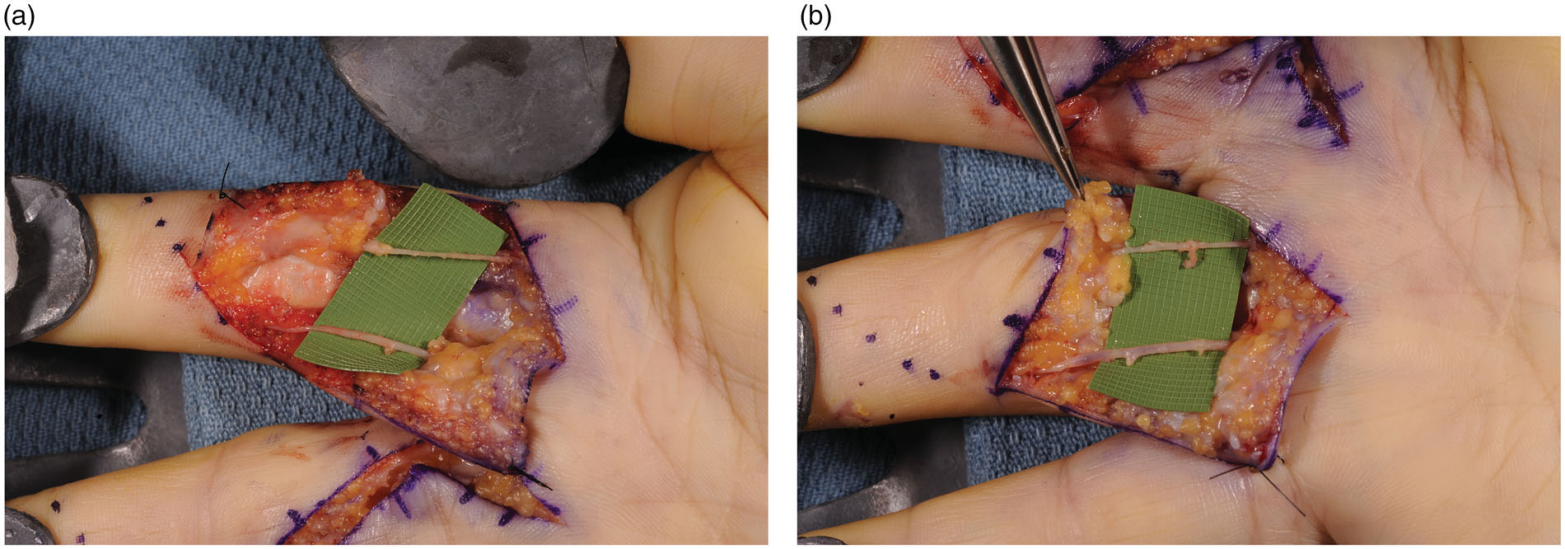
(a,b) Formal sympathectomies were performed on both the radial and ulnar neurovascular bundles with use of the operating microscope.

**Figure 4.**
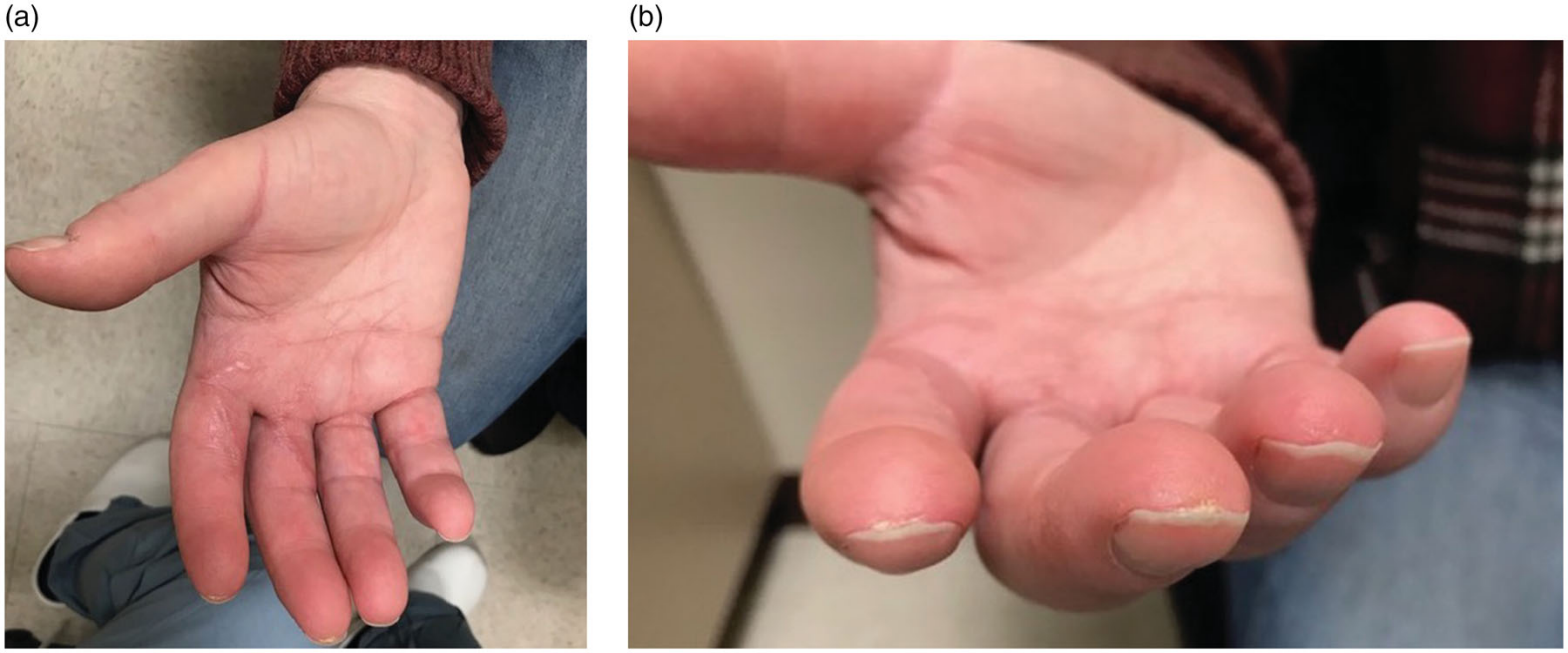
(a,b) Evidence of healed digital ulcerations at 3-month follow-up.

Surgery was performed under axillary nerve block with intravenous sedation. Bruner incisions were marked out and performed over the patient’s right index and middle fingers, extending from the distal palm to the respective proximal interphalangeal joints. Upon elevation of the skin flaps, numerous Pacinian corpuscles were noted amidst the volar subcutaneous tissue adjacent to the neurovascular bundles (Figure 2). Over the proximal phalanges of the index and middle fingers, between 10 and 12 corpuscles were appreciated per square centimeter. Formal sympathectomies, using an adventitial stripping technique, were performed on both the radial and ulnar neurovascular bundles with the use of the operating microscope (Figure 3(a,b)). At higher magnification, the extensive network of Pacinian tissue was appreciated surrounding the neurovascular bundles. The adventitia and much of the aforementioned Pacinian tissue were removed. The incisions were closed in standard interrupted fashion and the patient was placed in a forearm-based plaster splint. At the patient’s first postoperative visit 1 week later, his symptoms were noted to be markedly improved. At his 3-month follow-up appointment he was noted to be pain-free and his digital ulcerations were completely healed (Figure 4(a,b)).

**Figure 5.**
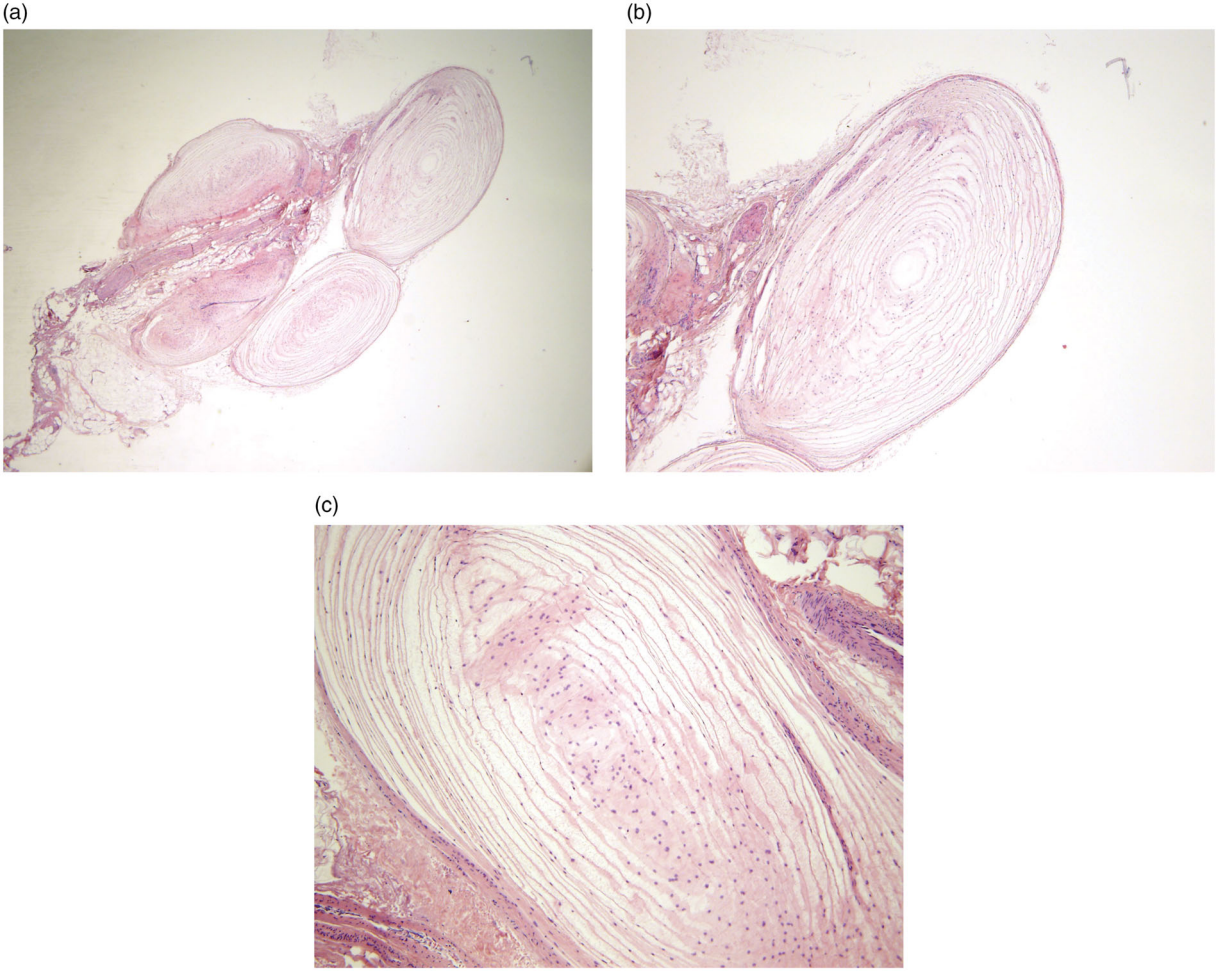
Cross-section of Pacinian corpuscle showing multi-lamellated connective tissue capsule at 2× (a), 4× (b), and 20**×** (c) magnification.

Tissue samples were sent to pathology for microscopic evaluation where the ultrastructures of the corpuscles were noted to be normal and not concerning for malignancy (Figure 5(a,b)). A pathologic diagnosis of Pacinian corpuscle hyperplasia was made.

## Discussion

The Pacinian corpuscle is a rapidly adapting mechanoreceptor, responding to the onset or cessation of pressure or vibration [2]. It is the only end-organ sensory receptor large enough to be visualized with the naked eye [2]. It consists of a single nerve fiber with a terminal non-myelinated portion enclosed within a multi-lamellated connective tissue capsule, akin to an onion, on cross-sectional histologic view (Figure 5(a,b)) [2,3,6]. The outer capsule is continuous with the perineurium of the adjacent digital nerve.

In the subcutaneous volar tissue of the hand, corpuscles can be found adjacent to the tendons or neurovascular bundles and are mostly clustered around the metacarpophalangeal joints and in the proximal phalanges of the three central digits [7]. Corpuscles can be differentiated from surrounding fat globules by their pearly-gray color and firm consistency. The average density is 3–5 corpuscles per cubic centimeter and up to 300 corpuscles is considered average for the human hand [3].

Pacinian pathology has been described previously, with proliferated corpuscles of normal or enlarged size along the neurovascular bundle constituting *hyperplasia*. Rhode and Jennings [8] characterized four different types of Pacinian hyperplasia, which are frequently cited in the other reports. Type A: a single, enlarged, subepineural corpuscle. Type B: a grape-like cluster of Pacinian corpuscles attached to the nerve by a fine filament. Type C: a series of enlarged corpuscles located in tandem, appearing as a branch of the nerve. Type D: many hyperplastic corpuscles located along the digital nerve as a single entity or in pairs. Reznik et al. [5] later modified this classification, believing that types C and D were the same.

The exact pathogenesis of Pacinian hyperplasia has not been fully elucidated. Approximately one-half of prior cases report a history of trauma to the affected area [9]. Manual labor where the digits are exposed to repeated microtrauma has also been implicated [10]. Previous cases were often confused with traumatic neuromas or peripheral nerve tumors [5]. Although the term ‘Pacinian hyperplasia’ has been used interchangeably with ‘Pacinian neuroma’ in the literature [6], they do not contain ultrastructure similar to neuroma [5]. Whereas traumatic neuromas exhibit histologic signs of Schwann cell proliferation or axons within dense fibrous tissue, these findings are not present in Pacinian hyperplasia [5]. The association with preceding trauma, therefore, may be misguided and over-reported. To date, there has been no association with malignancy.

In the current presentation, we report a finding of Pacinian hyperplasia in a patient with ischemic ulcerations in the setting of secondary Raynaud’s phenomenon. Pacinian corpuscles have close topographic relationships with the glomerular arteriovenous anastomosis [2], which has been previously described [11–13]. It is known that patients with Raynaud’s demonstrate heightened sympathetic vasoconstriction of the arteriovenous anastomoses [14]. Endothelial dysfunction is also implicated in secondary Raynaud’s and serves to further compound ischemic effects, particularly in the skin [14]. In their review of 33 cases, Imai et al. [2] believed that any biologic event that perturbed the relationship of the corpuscle and the arteriovenous anastomosis could result in the formation of new corpuscles. Early studies also showed that an interruption between the Pacinian corpuscle and the glomerular anastomosis could result in formation of a new receptor [12,13]. In experimental studies, Koshima et al. [15] observed morphological changes occurring in Pacinian corpuscles that correlated with the time of exposure to ischemia. In this way, Pacinian hyperplasia in our patient could represent a form of reactive hyperplasia secondary to chronic digital ischemia. We hypothesize that this may be the mechanism in our case, but future studies are required to further elucidate this as a potential ischemia-induced hyperproliferative pathway.

To our knowledge, Pacinian hyperplasia in a patient with Raynaud’s phenomenon has not been previously reported. In 1980, Levi and Cuni [11] described a case of Pacinian neurofibroma in a patient with ‘*Raynaud-like*’ symptoms that ultimately required amputation of the offending digit. Similarly, in their early investigation of the relationship between the digital arteriovenous anastomosis and the Pacinian corpuscle, Barbolini et al. [16] make mention of biopsying a Raynaud’s patient but do not provide details of the Pacinian structures. Herein, we have described a case of Pacinian hyperplasia found incidentally in a patient with Raynaud’s phenomenon and propose a potential mechanism of ischemia secondary to dysfunction (i.e. persistent vasoconstriction) of the glomerular arteriovenous anastomosis. Although the clinical relevance of this anatomic finding has not yet been determined, the surgeon may expect to encounter Pacinian hyperplasia in patients with long-standing vasospastic disease.

## Conclusion

Pacinian corpuscle pathology remains a rare clinical entity and may be an incidental finding during surgery of the hand. In patients with connective tissue disease or vasospastic disorders, dysfunction or interruption of adjacent glomerular arteriovenous anastomoses may elicit a hyperplastic response of Pacinian tissue and warrants further study.
